# Impact of pre-analytical factors on mycobacterium cultures contaminations rates in Burkina Faso, West Africa

**DOI:** 10.11604/pamj.2014.19.396.5551

**Published:** 2014-12-22

**Authors:** Antoinette Kaboré, Hervé Hien, Adama Sanou, Dézémon Zingué, Géraldine Daneau, Zakaria Ganamé, Moumini Nouctara, Mamoudou Ouédraogo, Oumarou Ouédraogo, Félicité Koutou, Michel Gomgnimbou, Nicolas Méda, Dorine Neveu, Sylvain Godreuil, Lassana Sangaré

**Affiliations:** 1 Department of transmissible diseases, Centre Muraz, Bobo-Dioulasso, Burkina Faso; 2 Institut de Recherche en Sciences de la Santé (IRSS), Direction Régionale de l'Ouest (DRO), Bobo-Dioulasso, Burkina Faso; 3 Centre Hospitalier Régional Universitaire (CHRU) de Montpellier, département de Bactériologie-Virologie, Montpellier, France; 4 Biomedical Science Department, Institute of Tropical Medicine, Antwerp, Belgium; 5 District Sanitaire de DAFRA, Bobo-Dioulasso, Burkina Faso; 6 University Montpellier 1, INSERM U1058 Infection by HIV and agents with mucocutaneous tropism from pathogenesis to prevention, Montpellier, France; 7 Bacteriology-Virology Department, CHU Yalgado Ouedraogo, Ouagadougou, Burkina Faso; 8 Departement of health sciences, University of Ouagadougou/Burkina Faso

**Keywords:** Sputum, storage, contamination

## Abstract

**Introduction:**

For a high quality level diagnosis, mycobacterium culture must comply with the pre-analytical and analytical conditions recommended by the WHO and the country National Tuberculosis Program (NTP). In this study, we determined whether temperature and duration of sputum storage were associated with culture contamination in Burkina Faso.

**Methods:**

Sputa were collected in 5 districts labs in Burkina Faso. Temperature and duration of sputum storage were recorded. After the collection, sputa were decontaminated using Petroff modified method, and the pellet was inoculated on LJ media and LJ media supply with 2% sodium pyruvate. Risk of culture contamination associated with temperature and duration of sputum storage was measured by Chi2 test and logistic regression.

**Results:**

Out of 404 specimens, 61% (246/404) were stored between 2 and 8°C, and 15% (61/404) were processed within three days. The global contamination rate was 24%, with only 8% for samples respecting WHO recommendations, up to 35% for others. Storage at room temperature was associated with a significantly higher risk of contamination compared to storage at 2-8°C (OR 2.24, p = 0.001, IC 95%).

**Conclusion:**

The recommendations about the temperature and the duration of sputum storage before cultures are not completely respected. This leads to high contamination rate of mycobacterium culture. It will be necessary to take logistics measures in peripherals health services or to develop more selective medium for mycobacterium culture in low income countries.

## Introduction

Despite encouraging progress in diagnosis and treatment, the global burden of tuberculosis (TB) remains huge with an estimated 6.1 million of new cases and 1.4 million of deaths in 2012. Besides, the multidrug resistant (MDR)-TB cases, essentially unchanged from 2011, are estimated to 450.000 cases. Globally, 3.6% of new TB cases and 20.2% of previously treated cases are estimated to have MDR-TB [1].

In Burkina Faso, a resource-limited country, TB prevalence is 82 cases per 100,000 people. Amongst those TB cases, mortality rate reaches 8.5 per 100,000, and HIV prevalence is 15 per 100 [1] In 2012, 68% (3583 cases) of all TB cases are new pulmonary TB cases with a positive smear microscopy for acid fast bacilli (AFB), and 14% (662 cases) are new pulmonary TB cases with negative smear microscopy for AFB[2]. The MDR-TB is a concern with an estimation of 154 cases among the notified cases and only 37 confirmed cases [2]. The microscopy for AFB with Ziehl Neelsen (ZN) staining is the cornerstone of tuberculosis diagnosis in low income countries [3, 4]. However, it has three major shortcomings: sensitivity is poor, requiring at least 5000-10000 bacilli/ml; it does not allow differentiation between atypical mycobacteria and complex tuberculosis mycobacteria; and it does not allow the diagnosis of MDR-TB [3, 4]. Recently, the Stop TB Strategy has been revised to improve diagnosis for TB smear negative microscopy and MDR-TB [5, 6]. This plan calls for accelerated access to mycobacterium culture [7]. Unfortunately, in Burkina Faso, as in most low- and some middle-income settings, quality of diagnosis by culture technique is hampered by high contamination rates. In a previous study conducted in the Centre MURAZ laboratory, culture contamination rate was superior to 25%, and numerous studies carried out in Africa and in Brazil on solid medium have reported culture contaminations rates between 10% and 37% [8–11] while the target range is 3-5% [12]. Those frequent contaminations reduce the proportion of interpretable results, and the value of culture as a diagnostic tool [8, 11].

Analytical and pre-analytical factors such as technique of sputum collection, storage temperature, transport conditions, duration between sample collection and processing, and lab methodology, can influence contamination rates [8, 10, 13, 14] To reduce the risk of culture contamination due to pre-analytical factors, the World Health Organization (WHO) and the Burkina Faso National TB program (NTP) have standardized procedures for sputum collection, storage, and transport condition [15, 16]. Briefly, if sputum collected in the field cannot be treated immediately, it should be kept in the refrigerator (at +2°C – +8°C), and transported in cool condition to the laboratory within 3-4 days after collection, according to WHO (or as fast as possible, according to NTP in Burkina Faso). Specimen should be collected in ad hoc containers, with a lid tightly secured, properly labelled and kept away from the sun and heat. These can be placed with ice packs in a special hermetic box to avoid leakage of contents, shocks and other incidents from ordinary handling practices; and transported to the reference laboratory [15, 16].

However, in Burkina Faso, we have no data on compliance with the WHO and NTP recommendations, and impact on the culture contaminations. In this study, we compute temperature of storage and transport; the duration between sputum collections and culture process, and investigated whether these variables were associated with culture contamination.

## Methods

### Sites and study population

Five sites were selected in Hauts-Bassins region in Burkina Faso: Centers for Diagnosis and Treatment (CDT) of Dafra, Dande, and Hounde, and the Centre Régional de Lutte Anti tuberculeuse (CRLAT) and the Pneumologie and Phtisiologie (PPH) service of Sourô Sanou University Hospital in Bobo-Dioulasso. Between March 2011 and February 2013, samples were prospectively collected from 427 adult pulmonary TB patients, with positive or negative AFB smear microscopy. The NTP technical guide was used for patient diagnosis. Socio-demographic data of the patients were recorded, and two sputa (2-3 ml each) were collected per patient at the screening site, and transported to Centre Muraz laboratory for processing. A written informed consent was obtained from all participants, and the study protocol was approved by the Institutional ethics committee of Centre Muraz, and by the National Ethical Review Board in Burkina Faso.

### Temperature and duration of sputum storage before the culture process

Sputum storage temperature (Room or refrigerator) was recorded at the screening site by means of a questionnaire. We considered the refrigerator temperature to be between +2°C and +8°C, and the room temperature 22°C and 35°C, the average minima and maxima ambient temperature reported by data from local weather agency of the Meteorology General Direction. Centre Muraz laboratory determined if sample was received on ice packs or not, and determined from the difference between dates of sputum collection and culture, the duration between the collection and the culture process.

### Sputum processing

The sputa were decontaminated using Petroff method. Briefly, sputum was decontaminated with 4% sodium hydroxide solution, and re-suspended in 1ml sterile distilled water; the obtained suspension was inoculated on two classical egg-based Löwenstein-Jensen (LJ) solid media (0.2ml suspension in each tube), and two LJ supplemented with 2% sodium pyruvate. Cultures were examined for contaminations and rapid growing bacteria the third and the seventh days after inoculation. Criteria for contaminated culture were: 1) any change in colour or consistency of culture media, 2) development of any liquid or film on the culture media, and/or 3) presence of non-mycobacterial colonies on culture media, on the base of colony morphology and/or ZN staining. Culture was contaminated if both samples of sputum were contaminated.

At days 14, 21, 28, 42, cultures were examined to assess positivity for Mycobacterium tuberculosis. Culture was positive if at least one of the two samples of sputum was positive. It was considered negative if there was no growth at day 90 for both samples of sputum.

To minimize culture contamination due to the analytical conditions in our laboratory, culture was routinely done under a class II biosafety cabinet (BSC II). Sterility of the culture medium was checked macroscopically after incubating every new batch at 37°C, during 72 hours. Sterile decontaminating solution was used. Distilled water used in culture was inoculated in parallel on sterile LJ medium, in order to check sterility.

### Statistical analysis

We determined the percentage of samples kept in refrigerator versus at room temperature, samples processed within 3 days after collection versus between 4-7 days, and versus more than 7 days. The Pearson Chi2 test was used for comparing contamination (presence versus absence). We estimated the risk of contamination associated with the determined pre-analytical factors with odds ratios and their 95% confidence interval using simple logistic regression with contamination as a dependant variable and the cofactors temperature and duration of storage as explanatory variables. Values of p < 0.05 were considered statistically significant. Data were analysed using the Stata software, version 12.0 (Stata Corp., College Station, TX, USA).

## Results

### Study population

A total of 427 patients were included in this study. Data of 23 patients were not analysed because cultures were not done in Centre MURAZ. Among the remaining patients, 27% (110/404) were women of 38 years old on average (range: 15-75); and 73% (294/404) were men of 41 years old on average (range: 15 - 90). For AFB at microscopy, 84% (339/404) of sputum specimens were positive and 16% (65/404) were negative. For the culture, 69% (279/404) were positive, 6.9% (28/404) were negative, and 24% (97/404) were contaminated. Among positive smear microscopy samples, 5/339 (1.5%) did not grow on culture. For the negative smear microscopy samples, 35% (23/65) were negative on culture, 40% (26/65) were positive and 25% (16/65) were contaminated.

### Compliance about temperature and duration of sputum storage

Globally, 61% (246/404) of sputa were stored in refrigerator, and 39% (158/404) at room temperature, data are showed in Table 1. All sputa (148) collected at CRLAT and PPH were kept in refrigerator, while all sputa (127) collected in remote centers as Dandé and Houndé were kept at room temperature. For the sputa collected at CDT of Dafra, 76% (98/129) were kept in refrigerator, and 24% (31/129) at room temperature, corresponding to sputa transferred from remote centers. Duration between collection and sputum processing for each site is shown in Table 1, using the following classification: < 3 days (recommended by WHO); between 4-7 days (still acceptable according to PNT), and > 7 days. Only 15% (61/404) of samples were processed within 3 days, and most of them 84% (51/61) were kept in refrigerator. For samples processed after respectively 4-7 days and more than 7 days, only 58% (61/105) and 56% (134/238) of them were kept in cool condition. All sputum samples were packed with ice-packs from each screening site to Centre MURAZ laboratory, as recommended.

**Table 1:** Sputum temperature and duration of storage according to screening sites

Screening sites	Storage Temperature	All sputum	≤ 3days	4-7days	> 7days
CRLAT	2-8°C	89	14	28	47
Dafra	2-8°C	98	6	24	68
	RT°	31	1	6	24
Dande	RT°	63	1	15	47
Hounde	RT°	64	8	23	33
PPH	2-8°C	59	31	9	19
All, n (%)	2-8°C	246 (61)	51 (13)	61 (15)	134 (33)
	RT°	158 (39)	10 (2.5)	44 (11)	104 (26)
**Total**		**404 (100)**	**61 (15)**	**105 (26)**	**238 (59)**

Number of samples for each site according to temperature and duration of storage.

% = n / total samples for that category. RT° = room temperature (minima and maxima average 22°C and 35°C respectively)

### Association between temperature and duration of sputum storage with culture contamination

**Figure 1:**
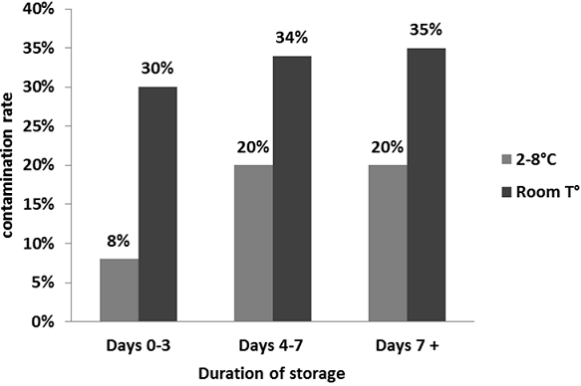
Histogram of contamination according to temperature and duration of sputum storage

Factors associated with contamination are shown in Table 2: contamination rate was significantly lower if sputum was stored in the refrigerator than at room temperature (17.5% (43/246) vs 34.2% (54/158) (p = 0.001). If sputum was processed within 3 days after collection, contamination rate was significantly lower than if processed between 4-7 days or >7 days (respectively 11.5% (7/61) vs 25.7% (27/105)( p = 0.097), and 26.5% (63/238), (p = 0.066)). Adjusted odd ratio, depending on temperature and duration of sputum storage before culture process, showed twice more risk of contamination (2.24; p = 0.001) for sputum kept at room temperature. Sputum kept at room temperature showed high contamination rate (30% or more) whatever the duration of sputum storage (Figure 1). In contrast, sputa kept in the refrigerator had lower contamination rate (8%) for quick process (= 3 days), than for longer duration of storage before process (20% for both 4-7 days and > 7 days; p = 0.129).

**Table 2:** Sputum temperature and duration of storage associated with contamination

Factors	Contamination rate% (n/total)	Crude OR [95%IC]	Adjusted OR [95%IC]	P-value
**Temperature of sputum storage**				
2-8°C	17.5 (43/246)	1	1	-
RT°	34.2 (54/158)	2.45 [1.54-3.90]	2.24 [1.40-3.60]	**0.001**
**Duration of sputum storage**				
**≤** 3 days	11.5 (7/61)	1	1	-
4-7 days	25.7 (27/105)	2.67 [1.08-6.57]	2.17 [0.87-5.43]	0.097
> 7 days	26.5 (63/238)	2.78 [1.20-6.42]	2.23 [0.95-5.23]	0.066

Contamination rate of culture from samples stored at 2-8°C or room temperature (RT, 22-35°C), and for samples received at reference lab for culture processing within 3 days, between 4 and 7 days, or more than 7 days

## Discussion

Microscopy for TB diagnostic has a limited sensitivity [3, 4]. Cultures can almost double the number of microbiologically-confirmed cases of TB [3]. Culture is even more important when dealing with surveillance, diagnosis and treatment of MDR-TB [3, 4]. However, contamination prevents culture from having a good diagnostic value. So contamination is currently challenging. Pre-analytical factors were described as influencing mycobacterium culture contamination rate. [8, 10, 11, 13, 14] In our study, we analysed two pre-analytical factors and evaluate their impact on culture contamination: temperature of sputum storage and transportation, and the duration of storage before culture process. Our results confirmed that temperature of storage was important, because contamination was 2.24 times more frequent when sputum was stored at room temperature versus in the refrigerator. Duration of storage was only a second factor, not significantly influencing. Samples processed before 3 days seemed less prone to contamination (11.5%), Compared with those processed between 4-7 days or after 7 days (25.7%, P= 0.097 and 26.5%, P= 0.066 respectively). The contamination rate seems to have achieved a maximal limit. Duration of storage had no impact when sputum was kept at room temperature, with high contamination rate even when quickly processed (30%). The most likely hypothesis is that growth of the commensal germs in the sputum is going to make scarce existing nutriments and to slow down even to stop microorganisms proliferation after three days of storage at room temperature.

Unfortunately, number of samples that do not respect the recommended maximum of 7 days of storage and the storage at 2-8°C was the majority (72%). Although all the screening sites were informed about NTP recommendations, i.e. storage in refrigerator and culture processing within 7 days (3 days according to WHO), we found that still 39% of sputa were kept at room temperature, and 59% were processed more than 7 days after collection. These results showed that recommendations were not correctly applied. The lack or the insufficiencies in logistics (refrigerators) and human's resources (biomedical technologists) were reported as the main reasons for this situation. For CDT of Dafra, sputa collected on study site were stored in refrigerator, but those received from remote centers, were stored at room temperature because these remote centers do not have laboratories nor refrigerators and generator to supply power. Sputum collected at CDTs of Dandé and Houndé were all kept at room temperature despite availability of laboratories and refrigerators on sites, because biomedical technologists prioritize storage of vaccines and reagents in refrigerators instead of samples. In fact, according to the good practice of laboratory, samples and reagents must not be kept in the same refrigerator because of the risk of reagent's contamination. This attitude can then be explained by the low availability of refrigerators. On the contrary, urban sites as CRLAT and PPH, encounter no logistic problems of conservation, so all sputa were kept in refrigerator after AFB test processing, before shipping to Centre MURAZ laboratory for culture.

Transport of sputa to the reference site must be done by biomedical technologist via public or personal mean of transport. Unfortunately, their number is insufficient and they cannot travel several times a week. Storage of sputum at room temperature favours the proliferation of commensal germs [4, 17], and then can increases the risk of culture contamination. A study from India evaluating impact of storage temperature showed a high contamination rate (18%) when samples were kept at room temperature for 7 days, and processed by using modified Petroff's method for decontamination [13] Lower contamination rate (5%) was obtained when samples were immediately processed after collection. Conclusion of this study is similar to our findings, i.e. that contamination rate increased when sputum were kept at room temperature. However, considering contamination rate obtained at room temperature after 7 days of storage, our contamination rate was twice higher than that of Paramasivan (34% vs 18%). One of the hypotheses to explain discrepancies is the difference of ambient temperature between regions, India and Burkina Faso: 30°C in India versus 35°C (average maxima) in Burkina Faso. The hotter weather at room temperature in Burkina Faso could engender a more important increase of commensals germs, and increase contamination rate. In addition, another factor could play a role: patients do not necessarily rinse mouth before sputum collection, and this factor is known to increase contamination rate [10, 11, 14]. Seen that the oral hygiene is insufficient in Burkina-Faso, one of the limits of this study is that no site recommended to the patients the mouth rinsing. This factor, could also explain the 8% contamination rate when sputa were kept in refrigerator before processing within 3 days, higher than the 3-5% expected [3, 12]. A slow proliferations of microorganisms in refrigerator, but not completely inhibited could explain the high contamination (20%) of sputum stored more than 3 days, even at refrigerator. Another factor that could explain this high contamination is keeping sputum first at room temperature during the microscopy processing. Those 2-4 hours could be enough for the commensal germs of the mouth and the pharynx, microorganisms multiplying within a few minutes, to double or triple the initial population, making decontamination procedure insufficient. The proportion of culture-negative samples despite positive smear microscopy (1.4%) showed that decontamination procedure was appropriate, and cannot be stronger.

## Conclusion

The recommendations about the temperature and the duration of sputum storage before cultures process are not completely respected. This leads to high contamination rate of mycobacterium culture and the global effort to control tuberculosis must consider this frequent problematic in low income countries. It will be necessary to supply refrigerators and generators in peripherals health services or to develop more selective medium for mycobacterium culture.
